# The Synergistic Relationship Between Atrial Fibrillation and Diabetes Mellitus: Implications for Cardiovascular and Metabolic Health

**DOI:** 10.7759/cureus.45881

**Published:** 2023-09-24

**Authors:** Maryam Mohsin, Hafiz Zeyad, Hareem Khalid, Abubakar Gapizov, Ruqiya Bibi, Yashkumar Girdharlal Kamani, Ahmed Rashid, Muhammad Shams, Faizan Khalid, Syeda Khan, Muhammad Waqas, Anzal Ishfaq, Ayele H Kebede, Muhammad Subhan

**Affiliations:** 1 Medicine, Lahore Medical and Dental College, Lahore, PAK; 2 Medicine and Surgery, Services Institute of Medical Science (SIMS), Lahore, PAK; 3 Medicine and Surgery, Services Hospital Lahore, Lahore, PAK; 4 Cardiac Surgery, Punjab Institute of Cardiology (PIC), Lahore, PAK; 5 Medical School, Lahore Medical and Dental College, Lahore, PAK; 6 General Surgery, American University of Antigua, St. John's, ATG; 7 Medicine and Surgery, Jinnah Hospital, Lahore, PAK; 8 Medical College, Allama Iqbal Medical College, Lahore, PAK; 9 College of Medicine, Cagayan State University College of Medicine, Tuguegarao, PHL; 10 General Practice, Yangtze University, Jingzhou, CHN; 11 Urology, Royal Bournemouth Hospital, Bournemouth, GBR; 12 Internal Medicine, King Edward Medical University, Lahore, PAK; 13 Medicine and Surgery, Dow University of Health Sciences, Karachi, PAK; 14 Internal Medicine, Iqra Medical Center and Maternity Home, Karachi, PAK; 15 Internal Medicine, Jinnah Sindh Medical University, Karachi, PAK; 16 Internal Medicine, Mayo Hospital, Lahore, PAK; 17 Internal Medicine, Kersa Primary Hospital, Asela, ETH; 18 Internal Medicine, Allama Iqbal Medical College, Jinnah Hospital, Lahore, PAK; 19 Medicine and Surgery, Al Barkat Health Care and Collection Centre, Lahore, PAK

**Keywords:** ecg holter monitoring, type 2 diabetes mellitus, treating patients with cardiovascular maladies, metformin in heart disease, glycated hemoglobin (hba1c), structural heart procedures, adult cardiac disease, diabetes and heart complications, afib and dm, new onset afib

## Abstract

Type 2 diabetes mellitus (T2DM) and atrial fibrillation (AF) are widespread chronic conditions that profoundly impact public health. While the intricate mechanisms linking these two diseases remain incompletely understood, this review sets out to comprehensively analyze the current evidence about their pathophysiology, epidemiology, diagnosis, prognosis, and treatment. We reveal that T2DM can influence the electrical and structural properties of the atria through multiple pathways, including oxidative stress, inflammation, fibrosis, connexin remodeling, glycemic variability, and autonomic dysfunction. Moreover, it significantly influences AF's clinical course, elevating the risk of heart failure, stroke, and cardiovascular mortality. Our review also explores treatment options for individuals with T2DM and AF, encompassing antidiabetic and antiarrhythmic drugs and non-pharmacological interventions, such as cardioversion catheter ablation and direct current cardioversion. This review depicts an insight into the clinical interplay between T2DM and AF. It deepens our comprehension of the fundamental mechanisms, potential therapeutic interventions, and their implications for patient care. This comprehensive resource benefits researchers seeking to deepen their knowledge in this domain. Ultimately, our findings pave the way for more effective strategies in managing AF within the context of T2DM.

## Introduction and background

Type 2 diabetes mellitus (T2DM) poses a serious global health challenge, affecting millions with chronic metabolic disruptions [1]. Raised blood glucose levels characterize this condition due to insulin resistance and insufficient insulin secretion [1]. T2DM has emerged as a leading contributor to cardiovascular and cerebrovascular complications, including atherosclerosis, peripheral artery disease (PAD), acute myocardial infarction (AMI), stroke, and peripheral neuropathy (PN) [2]. In parallel, AF is a cardiac arrhythmia characterized by irregular and rapid atrial activity [3]. AF combined with DM poses significant risks, notably increasing the likelihood of thromboembolic events, heart failure, and mortality [3,4]. AF can lead to blood clot formation, exacerbated by DM's metabolic and vascular changes, heightening the risk of strokes and embolic events [3-5].

In addition, this combination worsens heart failure due to impaired cardiac function and circulation [2,3]. Ultimately, the coexistence of AF and DM significantly threatens patient health and longevity, emphasizing the need for effective management and prevention strategies [3,4]. The association between T2DM and AF has been recognized for decades, yet the evidence needs to be more consistent and conclusive [4]. The first epidemiological study reporting a positive correlation between T2DM and AF was published in 1974 by Kannel et al. [1,3]. Importantly, individuals with T2DM exhibit a higher incidence of AF than their non-diabetic counterparts. However, this association's intricate mechanisms still need to be understood [3,5]. This review delves into the clinical interplay between T2DM and AF to untangle this complex relationship. Diagnosing AF in T2DM patients can be challenging, as both conditions may present nonspecific or asymptomatic features [3]. Therefore, it is crucial to employ appropriate methods and criteria to detect AF in this population [3]. The diagnosis of AF typically involves electrocardiography (ECG), Holter monitoring, or implantable devices [3]. However, these methods may have limitations regarding sensitivity, specificity, cost, or availability [3].

Consequently, new technologies and biomarkers are being developed and tested to enhance the AF diagnosis in T2DM patients [3]. T2DM significantly worsens the prognosis of AF [1,3]. Age, gender, ethnicity, comorbidities, glycemic control, and anticoagulation therapy influence AF outcomes in T2DM patients [1,3]. Hence, meticulous monitoring and comprehensive management of these factors are imperative for individuals with both conditions [1,3]. This study comprehensively examines the link between T2DM and AF, covering epidemiology, mechanisms, diagnosis, prognosis, and treatment. It provides valuable insights and strategies for managing AF in individuals with T2DM. By exploring the pathophysiological realm, we scrutinize the mechanisms connecting T2DM and AF, including oxidative stress, inflammation, fibrosis, connexin remodeling, glycemic variability, and autonomic dysfunction [5,6]. Moving forward, we navigate the epidemiological landscape, unpacking the evidence that speaks to the association between T2DM and AF incidence, recurrence, and complications [7-9].

Furthermore, we pivot to the diagnostic sphere, delving into the methods and criteria for detecting AF in T2DM patients [7,8]. As we traverse the territory of prognostication, we unveil the factors and outcomes that shape AF's trajectory in the presence of T2DM [5-10]. Ultimately, we take an encompassing view of therapeutic approaches, exploring options that span antidiabetic and antiarrhythmic drugs alongside non-pharmacological interventions, such as cardioversion catheter ablation and direct current cardioversion [11-12]. By synthesizing this multifaceted evidence, we aim to enhance the understanding and management of patients grappling with the dual burdens of T2DM and AF.

## Review

Methodology

The methodology for this comprehensive review involved a systematic search of relevant literature in databases, such as PubMed, MEDLINE, Scopus, Web of Science, and Google Scholar. A list of keywords, including "type 2 diabetes mellitus," "atrial fibrillation," "AF," "diabetes," "pathophysiology," "epidemiology," "diagnosis," "prognosis," and "treatment," was employed. Boolean operators were used to refine searches. Inclusion criteria comprised articles discussing the relationship between T2DM and AF across various aspects, including pathophysiology, epidemiology, diagnosis, prognosis, and treatment. The study types considered encompassed systematic reviews, meta-analyses, randomized controlled trials (RCTs), observational studies, clinical trials, and experimental studies. Studies that do not depict the relationship between T2DM and AF or if they focused solely on one aspect without contributing substantively to the review's objectives were excluded. Non-English language articles were excluded for consistency.

Two separate evaluators screened articles based on their titles and abstracts, and full-text articles meeting the inclusion criteria underwent additional assessment. Data extraction included study titles, authors, publication years, study designs, methodologies, sample sizes, demographic characteristics, key findings, and methods used in pathophysiological investigations, diagnosis, treatment, and prognosis assessment. A narrative synthesis approach organized and presented the extracted data coherently, providing insights into the current knowledge regarding T2DM and AF and identifying research gaps. Quality assessment tools, such as the Newcastle-Ottawa scale for observational studies and the Cochrane Collaboration's tool (CCT) for RCTs, were utilized to evaluate potential sources of bias within studies. This comprehensive methodology ensures a well-structured analysis of the complex relationship between T2DM and AF across multiple dimensions, laying a robust foundation for subsequent discussions and research in the comprehensive review.

Discussion

This comprehensive review explores the intricate clinical connection between T2DM and AF. T2DM and AF are two prevalent chronic conditions contributing significantly to global health burdens [1]. Epidemiologically, both T2DM and AF have shown remarkable prevalence [1,2]. T2DM, in particular, has witnessed a surge, with increasing global incidence [1]. The clinical presentation of T2DM and AF can be diverse and challenging, and patients may exhibit nonspecific features, fatigue, shortness of breath, increased urination, thirst, palpitations, general malaise, chest discomfort, and dizziness or asymptomatic features, underscoring the need for precise diagnostic criteria [3]. Risk factors, including age, obesity, hypertension, and genetic predisposition, further complicate the clinical picture [1,3]. The management of individuals with coexisting T2DM and AF demands a multifaceted approach. Pharmacological interventions involve antidiabetic drugs, antiarrhythmic drugs, and anticoagulants [3]. Non-pharmacological interventions include cardioversion catheter ablation, lifestyle modifications, and surgical procedures [3]. As we delve deeper into this discussion, we will explore the pathophysiological mechanisms, clinical associations, and therapeutic recommendations linking T2DM and AF.

Pathophysiological Associations of Diabetes and AF

The review's findings demonstrate that T2DM can influence atrial electrophysiology and structure through diverse pathways [5]. These pathways encompass oxidative stress, inflammation, fibrosis, connexin remodeling, glycemic variability, and autonomic dysfunction, culminating in alterations to the atrial action potential duration, refractoriness, conduction velocity, and dispersion [6,7]. These alterations predispose the atria to abnormal impulse generation and conduction, contributing to AF susceptibility [5-7]. These findings concur with previous studies highlighting the role of metabolic stress in driving atrial remodeling and increasing the susceptibility to AF [5-6]. However, the precise mechanisms by which T2DM influences atrial ionic currents and calcium homeostasis remain enigmatic. Some studies suggest that T2DM may impede the function of the sodium-potassium pump (Na+/K+-ATPase), potentially resulting in intracellular sodium accumulation and calcium overload [11-13].

In addition, T2DM might impact the expression and phosphorylation of the L-type calcium channel (LTCC), potentially leading to reduced calcium influx and contractility [12,13]. Furthermore, T2DM could modulate the activity and expression of various potassium channels, including the inward rectifier potassium channel (IK1), transient outward potassium channel (Ito), ultra-rapid delayed rectifier potassium channel (IKur), and acetylcholine-activated potassium channel (IKACh) [11-15]. These alterations might induce changes in atrial action potential morphology and duration [12]. Further comprehensive studies are needed to clarify the molecular processes underpinning T2DM-induced atrial electrical remodeling and identify potential targets for pharmacological intervention [13,14]. The review also establishes that T2DM is associated with a 1.4- to 1.6-fold heightened risk of developing AF, an elevated risk that persists independently of other common risk factors [3].

Furthermore, T2DM can significantly influence AF's clinical trajectory and outcomes, contributing to an augmented risk of stroke, heart failure, and cardiovascular mortality [3]. These findings align with prior research illustrating the adverse effects of T2DM on AF incidence, recurrence, and complications [1,3]. Nonetheless, the exact reasons for this amplified risk still need to be understood. Plausible explanations encompass subclinical or overt cardiovascular diseases, such as coronary artery disease and the thickening of the left ventricle, diastolic dysfunction, or valvular disease, which may predispose T2DM patients to AF [11-16]. Another contributing factor is the increased thromboembolic risk in T2DM patients due to hypercoagulability, endothelial dysfunction, platelet activation, or inflammation [14-18].

**Figure 1:**
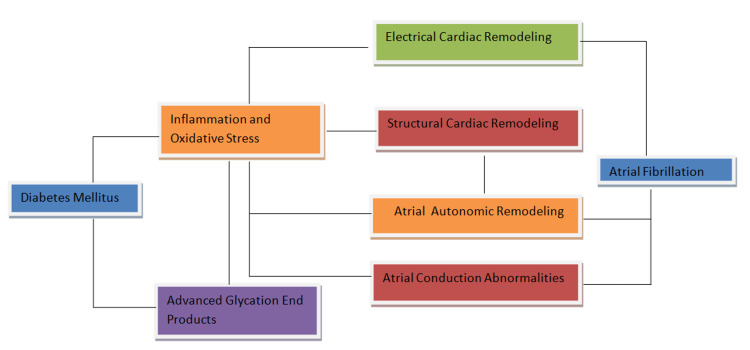
Pathophysiological pathways interlinking T2DM and AF T2DM: type 2 diabetes mellitus; AF: atrial fibrillation [5] Image credit: Ruqiya Bibi

Moreover, T2DM's influence on the response to antiarrhythmic drugs or non-pharmacological interventions, such as catheter ablation or cardioversion, adds further complexity to this association [15-19]. More extensive studies are warranted to unravel the causal relationship between T2DM and AF and to identify potential modifiers or mediators of this complex connection. The pathophysiological mechanisms that initiate AF in T2DM are shown in Figure 1.

Clinical Correlation of DM and AF

DM and AF represent two common chronic conditions with substantial impacts on the health and quality of life of millions worldwide [1]. DM, a metabolic disorder characterized by high blood sugar levels resulting from either insulin resistance or a lack of insulin production, intertwines with AF, a cardiac arrhythmia marked by irregular and rapid heartbeats resulting from abnormal electrical impulses in the atria [1]. Both conditions independently confer elevated risks of cardiovascular complications, including stroke, heart failure, and mortality [3]. Several studies have depicted the association between DM and AF and the potential mechanisms and risk factors underpinning this relationship. However, this association is complex and multifactorial, and several aspects remain unclear or controversial. This narrative review discusses issues and challenges in the clinical correlation of DM and AF, such as the effect of glycemic control on the development and progression of AF in patients with DM, as well as the optimal target range and measurement methods for glycemic control [4]. Glycemic control is crucial in managing DM to avoid long-term microvascular and macrovascular complications [4]. However, the impact of glycemic control on AF risk and outcomes is not well established. Some studies have suggested that poor glycemic control may increase the risk of AF by promoting oxidative stress, inflammation, fibrosis, and autonomic dysfunction in the atria [7-9]. Other studies have found no significant association between glycemic control and AF incidence or recurrence [10-11].

Moreover, the optimal level of glycemic control for AF prevention or treatment needs to be clarified. Some guidelines recommend a target hemoglobin A1c (HbA1c) level of <7% for most patients with DM [4-6], while others suggest individualized targets based on patient characteristics and preferences [7,8]. The best method for measuring glycemic control in patients with DM and AF is still being determined [8]. HbA1c test depicts the average blood sugar level over the previous two to three months, but it may be affected by other factors, such as anemia, hemolysis, or chronic kidney disease [4,8,10]. Other methods, such as self-monitoring blood glucose or continuous glucose monitoring, may provide more accurate and timely information on glycemic fluctuations and variability, influencing AF risk and outcomes [4,7,10].

Numerous studies have sought to unravel the association between DM and AF alongside the potential mechanisms and risk factors underpinning this relationship [10,11]. Ostergren et al. conducted a retrospective study involving 793 patients with or without hypertension and T2DM and identified diabetes as an independent predictor of AF, particularly among women [20]. This study was conducted in Skara, Sweden, involving 1,739 participants and explored AF prevalence in those with hypertension and T2DM [20]. They categorized subjects into groups: hypertension-only, both hypertension and T2DM, T2DM only, and normotensive controls [20]. AF prevalence, adjusted for age, was 2% (hypertension-only), 6% (combined hypertension and T2DM), 4% (T2DM-only), and 2% (controls) [20]. Adjusted odds ratios (OR) were 0.7 (hypertension), 3.3 (combined hypertension and T2DM), and 2.0 (T2DM) [20]. The association remained when considering cardiovascular risk factors and body mass index (BMI) but weakened with ischemic ECG [20]. It disappeared when adjusting for insulin resistance [20]. In summary, AF is linked to combined hypertension and T2DM, possibly influenced by insulin resistance [20].

Dublin et al.'s population-based case-control study of 1,410 patients with new-onset AF and 2,203 individuals without AF reported a 40% elevated risk of AF associated with diabetes, even after accounting for other confounding factors [21]. Among individuals with AF, those with treated diabetes had a 1.40-fold higher risk of AF compared to those without diabetes [21]. The risk increased by 3% per additional year of diabetes duration. When compared to those without diabetes, adjusted ORs for AF risk were 1.06 for average hemoglobin A1c levels ≤7, 1.48 for A1c levels >7 but ≤8, 1.46 for A1c levels >8 but ≤9, and 1.96 for A1c levels >9 [21]. In summary, diabetes was linked to a higher AF risk, especially with longer diabetes duration and poorer glycemic control, emphasizing the need for research into AF risk reduction for people with diabetes [21].

Furthermore, Alijla et al.'s systematic review and meta-analysis, which included eight cross-sectional and 14 longitudinal studies, highlighted that diabetes increased the AF risk by 39% in cross-sectional studies and 28% in longitudinal studies [22]. The study by Seyed Ahmadi et al. was a cohort study of 421,855 patients with T2DM and 2,131,223 controls [23]. The research revealed that people with T2DM faced a 35% higher risk of developing AF than the control group [23]. The excess risk for AF increased with renal complications or poor glycemic control [23]. People with T2DM who maintained reasonable glycemic control (HbA1c ≤ 6.9% or ≤ 52 mmol/mol) and had normoalbuminuria experienced a slight elevation in risk [23]. In addition, in a study conducted by Nichols et al., the research involved a study population comprising 10,213 individuals from a health maintenance organization (HMO) diabetes registry and an additional 7,159 patients who joined the registry by December 31, 2004 [24]. These individuals were matched with patients without diabetes based on their year of birth and sex [24]. The study tracked these patients until various endpoints, including death and departure from the health plan [24]. The average age of the study population was 60 years, and 46% were women [24]. The study's key findings indicated that diabetes was associated with a 40% higher prevalence of AF and a 26% higher incidence of AF, even after adjusting for potential confounding factors [24]. Furthermore, the study revealed that the elevated risk of AF linked to diabetes was more pronounced in women than in men and was more prominent in younger age groups than older ones [24]. In conclusion, the study established that diabetes independently contributes to an increased prevalence and incidence of AF [24].

In a study conducted by Schoen et al., they found that T2DM was linked with a 40% increased risk of incident AF in women, independent of other cardiovascular risk factors and biomarkers [25]. This prospective cohort study included 34,309 women from the Women's Health Study who were initially free of AF and followed for a median of 16 years [25]. The analysis, adjusted for various factors, including age, race, lifestyle factors, and biomarkers, showed that the incidence rate of AF was higher in women with diabetes (5.4 per 1,000 person-years) compared to those without diabetes (3.6 per 1,000 person-years) [25]. In addition, the study found a positive association between glycated hemoglobin (HbA1c) levels and AF risk, indicating that poor glycemic control might contribute to AF development [25]. The study concluded that T2DM independently increased the risk of incident AF in women and that suboptimal diabetes management could elevate AF risk [25]. These results underscore the significance of efficiently controlling diabetes and addressing its complications as a potential strategy to delay or mitigate the occurrence of AF and its related repercussions [25]. Regarding differences in this study population, the study specifically focused on women from the Women's Health Study who were initially free of AF [25]. It did not delve into diabetes control or comorbidities in detail but did highlight the association between poor glycemic control (as indicated by HbA1c levels) and an increased risk of AF [25].

The study by Xiong et al. conducted a systematic review and meta-analysis employing machine learning techniques to identify relevant studies investigating the link between DM and new-onset AF [26]. This study used machine learning to streamline publication screening, identifying 29 relevant studies from a pool of 4,177 articles [26]. The meta-analysis showed that DM increased AF risk by approximately 49%, even after adjusting for hypertension, obesity, and heart disease with a risk of 23% [26]. This risk remained consistent across DM subtypes, with women having a 24% higher risk than men [26]. In addition, the study noted a growing trend in AF risk among DM patients over time [26]. In summary, machine learning aids efficient study selection and confirms DM, especially in women, as a substantial independent AF risk factor [26]. Wang et al.'s prospective cohort study of 14,286 participants revealed that diabetes was linked to a 40% increased risk of incident AF [27]. Some of these critical studies with their results are summarized in Table 1.

**Table 1:** Characteristics of studies on the association between T2DM and AF T2DM: type 2 diabetes mellitus; AF: atrial fibrillation; ECG: electrocardiography; OGTT: oral glucose tolerance test; GH: general health

Authors	Type of study	Patients	AF diagnosis	DM diagnosis	Mean age
Ostergren et al. [20]	Retrospective study	793 patients with or without hypertension and T2DM	ECG	Self-report oral glucose tolerance test (OGTT)	61 ± 31.5 in men 60 ± 12.5 in women
Dublin et al. [21]	Population-based case study	1,410 patients with new-onset AF and 2,203 people without AF	GH electronic data	N/A	74 (66-80)
Alijla et al. [22]	Systematic review and meta-analysis	Eight cross-sectional and 14 longitudinal studies involving patients with AF and diabetes or glucose metabolism disorders	ECG or Holter monitoring	Fasting glucose/OGTT	Varies by study
Seyed Ahmadi et al. [23]	nationwide cohort study	406,436 individuals with T2D and 2,024,380 matched controls without diabetes from Sweden	ECG	OGTT	62.7 (for cases) 61.3 (for controls)
Nichols et al. [24]	Retrospective study	7,372 patients with or without Diabetes	NA	NA	58.4 ± 11.5
Schoen et al. [25]	Cox proportional- hazards model	34,720 females with or without Diabetes	ECG	Hb1AC	54.1 (48.9-62.1)
Wang et al. [27]	Prospective cohort study	14,286 individuals with or without diabetes from the Atherosclerosis Risk in Communities (ARIC) study	ECG	Fasting glucose greater than 126 mg/dL or non-fasting glucose greater than 200mg/dl	54 ± 6

Treatment Recommendations

The therapeutic implications for AF and DM are multifaceted, demanding a nuanced, individualized approach to mitigate adverse outcomes [23-25]. These implications encompass several critical aspects:

Anticoagulation: Anticoagulation is pivotal in stroke prevention for AF patients [27,28]. Nonetheless, the optimal anticoagulant choice and dosage can vary based on the severity of DM and other factors, such as renal function, bleeding risk, and drug interactions [23-29]. Some studies suggest potential advantages of non-vitamin K antagonist oral anticoagulants (NOACs) over warfarin in AF patients with DM, although findings are inconclusive [28,29]. NOACs depicted a lower risk of significant bleeding and similar effectiveness in reducing stroke risk compared to warfarin in AF patients with DM, suggesting that they may be preferable in this population [29-32]. NOACs provide a more predictable anticoagulant effect without frequent monitoring [32-34]. In a meta-analysis comprising 12 RCTs and a total of 57,491 patients diagnosed with both AF and DM, it was determined that NOACs exhibited a reduced risk of stroke or systemic embolism, major bleeding, intracranial hemorrhage, and all-cause mortality when compared to the use of warfarin [32]. However, a subgroup analysis of the Randomized Evaluation of Long-Term Anticoagulation Therapy (RE-LY) trial found no significant difference in the efficacy and safety outcomes between dabigatran and warfarin in AF patients with DM [32]. Consequently, anticoagulation therapy should be tailored to each patient's unique characteristics and preferences while regularly monitoring for efficacy and adverse events [29-35].

Rate control: For most AF patients, rate control is the preferred strategy due to its potential to enhance symptom management, improve quality of life, and reduce the risk of tachycardia-induced cardiomyopathy [33]. However, the optimal target heart rate and choice of rate control agents may vary based on the presence and severity of DM and other comorbidities, such as heart failure, hypertension, and ischemic heart disease [34,35]. Beta blockers, for example, have shown potential benefits on glycemic control and cardiovascular outcomes in AF patients with DM, but individualization is crucial [35-38]. A systematic review encompassing 11 RCTs and a participant pool of 1,378 individuals diagnosed with AF and DM revealed that beta blockers outperformed calcium channel blockers or digoxin in terms of reducing resting and exercise-induced heart rates [25]. Using beta blockers during pregnancy is a complex medical decision requiring consultation with a healthcare provider [25]. Beta blockers are medications for various conditions, including high blood pressure and heart arrhythmias [25,34-38].

While they can benefit some pregnant individuals, they have potential risks [25,38]. Studies suggest a slight increase in fetal complications, including conditions, such as reduced birth weight and premature birth. When beta blockers are used during pregnancy [34-38], babies born to mothers on beta blockers may experience temporary symptoms, such as slow heart rate and low blood sugar [34-38]. Importantly, beta blockers can mask symptoms of hypoglycemia in pregnant individuals with diabetes [32-38]. When deciding whether to use beta blockers during pregnancy, healthcare providers consider the specific medical condition, its severity, and the risks and benefits to both mother and fetus [32-38]. Close monitoring and individualized care are essential to ensure the well-being of both [32-38]. Therefore, careful selection and dose adjustment of beta blockers are warranted in this population [25,32-38].

Rhythm control: Rhythm control presents an alternative strategy for AF patients experiencing persistent symptoms or having contraindications to rate control [37-39]. Rhythm control strategies play a pivotal role in managing AF, aiming to restore and maintain normal sinus rhythm (NSR) in patients [37,38]. Among the approaches used for rhythm control, pulmonary vein isolation (PVI), atrioventricular (AV) nodal ablation, and antiarrhythmic drugs stand out as noteworthy interventions [9,40-42]. PVI entails electrical disassociation of the pulmonary veins from the left atrium since these veins frequently generate abnormal electrical signals that initiate AF [42-44]. This procedure commonly uses catheter-based techniques, such as radiofrequency or cryoablation [42-44]. During PVI, the goal is to create scar tissue around the pulmonary veins, interrupting the abnormal electrical pathways responsible for AF [42-44]. PVI has been established as an effective rhythm control strategy, particularly for paroxysmal AF [42-44]. However, its success rate may vary depending on patient characteristics, AF duration, and the ablation technique employed [41-44].

Careful patient selection and a comprehensive evaluation by cardiac electrophysiologists are crucial for achieving optimal outcomes with PVI [9,41-44]. AV nodal ablation may be considered in some cases, especially when other rhythm control strategies are ineffective or contraindicated [9,45-48]. This procedure involves the deliberate destruction or ablation of the AV node, responsible for transmitting electrical signals between the atria and ventricles [9,48]. Following AV nodal ablation, the patient loses AV conduction, resulting in permanent pacemaker implantation to ensure adequate ventricular pacing [9,48]. AV nodal ablation effectively controls heart rate but does not restore NSR [9,48]. Therefore, it is typically reserved for patients with refractory AF, where the focus is primarily on rate control [9,48]. While AV nodal ablation can alleviate AF symptoms, it does not eliminate the arrhythmia [38,39]. Flecainide is an antiarrhythmic medication that plays a role in rhythm control by suppressing abnormal electrical signals in the heart and promoting NSR [38,39]. It is often used in patients with symptomatic AF, particularly in cases of paroxysmal AF [38,39]. Flecainide works by inhibiting sodium channels in cardiac cells, slowing conduction through the atria, and reducing the likelihood of AF episodes [38,39]. However, its use requires careful consideration, as it may be contraindicated in individuals with certain cardiac conditions or structural heart abnormalities [38,39,44].

Moreover, pro-arrhythmia with flecainide is risky and can potentially induce or worsen arrhythmias [38,39,44]. Therefore, patients prescribed with flecainide should be closely monitored, and its use should be tailored to the individual's specific clinical situation [44]. However, the efficacy and safety of rhythm control agents may fluctuate depending on the presence and severity of DM and other factors, such as structural heart disease, electrolyte imbalance, and drug interactions [38,39,44]. Amiodarone, in particular, has been suggested to offer lower recurrence rates and fewer adverse events in AF patients with DM [38,39]. A retrospective cohort study comprising 1,223 patients with both AF and DM showed that amiodarone was linked to a reduced risk of AF recurrence following electrical cardioversion compared to sotalol or propafenone [38,39]. However, amiodarone may also cause thyroid dysfunction or pulmonary toxicity, adversely affecting glycemic control or respiratory function [38,39]. A systematic review and meta-analysis of 11 RCTs involving 1,378 patients diagnosed with AF and DM determined that beta blockers outperformed calcium channel blockers or digoxin in reducing heart rate, both at rest and during exercise [38]. This study supports using beta blockers as the first-line agents for rate control in AF patients with DM, although they may also increase the risk of hypoglycemia or mask its symptoms [38]. A thorough evaluation of the patient's risk-benefit profile should guide rhythm control therapy, potentially in combination with anticoagulation when indicated [38,39,44].

Lifestyle modification: Lifestyle modification is pivotal in managing AF and DM [45]. It can enhance symptom management, glycemic control, cardiovascular risk factors, and overall quality of life [45,46]. Key aspects encompass weight loss, exercise, dietary modification, smoking cessation, alcohol reduction, stress management, and sleep hygiene [46,47]. These lifestyle changes can yield significant benefits, including reduced AF burden and improved glycemic control [45-48]. A prospective cohort study involving 355 overweight or obese patients with symptomatic AF found that a structured weight management program resulted in a dose-dependent reduction in AF symptom burden and severity over five years [41]. This study indicates that weight loss may be a beneficial non-pharmacological intervention for improving symptom management and quality of life in AF patients with DM [41]. An RCT involving 150 patients with poorly controlled T2DM found that a lifestyle intervention consisting of dietary counseling, physical activity promotion, and behavioral therapy led to a greater improvement in glycemic control than usual care over 12 months [49]. This study demonstrates that lifestyle modification may be an effective strategy for achieving glycemic control in patients with DM and potentially reducing the risk of AF [49,50].

Antidiabetic therapy: Antidiabetic therapy is essential in managing DM and its complications [51]. However, some antidiabetic drugs may also have beneficial or detrimental effects on AF occurrence or management [51]. Therefore, the choice of antidiabetic therapy should consider not only the glycemic efficacy but also the potential impact on AF risk or treatment [52]. Some antidiabetic drugs that have shown promising results in terms of atrial remodeling and AF prevention or treatment include metformin, thiazolidinediones (TZDs), sodium-glucose co-transporter-2 (SGLT-2) inhibitors, glucagon-like peptide-1 (GLP-1) receptor agonists, and dipeptidyl peptidase-4 (DPP-4) inhibitors [51,52]. However, the current evidence on the efficacy and safety of these drugs for AF management in T2DM patients remains limited and inconclusive [51,52]. Rigorous RCTs must confirm their anti-AF effects and determine optimal dosages [53-55]. In addition, it is essential to remain mindful of the potential adverse effects of some antidiabetic drugs on cardiac function or arrhythmogenesis, which may necessitate individualized selection based on the patient's characteristics, preferences, and comorbidities [53-55]. For example, metformin may cause lactic acidosis in patients with renal impairment or heart failure [51], TZDs may increase the risk of heart failure or ischemic events in patients with cardiovascular disease [53], and SGLT-2 inhibitors may cause volume depletion or ketoacidosis in patients with dehydration or insulin deficiency [54-56].

In a post-hoc analysis of two RCTs involving individuals with T2DM at high cardiovascular risk, semaglutide, designed to lower blood sugar levels, demonstrated significant benefits in reducing the incidence of first-time strokes compared to a placebo [57]. This reduction in stroke risk was primarily attributed to semaglutide's ability to prevent strokes caused by the blockage of small blood vessels in the brain, known as small-vessel occlusion strokes [57]. Importantly, semaglutide's protective effect against strokes was consistent whether or not participants had a prior history of stroke at the start of the trials [57]. In addition, it is noteworthy that semaglutide did not pose an increased risk of major adverse cardiovascular events, such as heart attacks or deaths, for participants, regardless of their prior stroke history [57]. These findings suggest a potential role for semaglutide in stroke prevention among individuals with T2DM at high cardiovascular risk, particularly in reducing small-vessel occlusion strokes without elevating overall cardiovascular risks [57].

Therefore, careful monitoring and dose adjustment of antidiabetic drugs is warranted in AF patients with DM [51-57]. The intricate interplay between T2DM and AF presents a multifaceted challenge in clinical management [51-57]. While the pathophysiological associations between these two conditions continue to emerge, it is evident that T2DM significantly influences atrial electrophysiology and structure, predisposing individuals to AF [51-57]. This heightened risk translates into adverse clinical outcomes, emphasizing the need for comprehensive management strategies [51-57]. The therapeutic landscape encompasses a range of options, from anticoagulation and rate control to rhythm control and lifestyle modifications [51-57]. Individualized treatment plans that consider each patient's unique characteristics and preferences are crucial to achieving optimal outcomes [51-57].

Moreover, ongoing research into the impact of antidiabetic drugs on AF management holds promise but requires further investigation to clarify their efficacy and safety profiles. Ultimately, the holistic care of patients with coexisting T2DM and AF demands a collaborative effort between healthcare providers to mitigate adverse outcomes and improve these individuals' quality of life. As our understanding of the intricate relationship between T2DM and AF continues to evolve, so will our approaches to diagnosis, prevention, and treatment, offering hope for better outcomes and improved lives for those affected by these prevalent chronic conditions.

Limitations

This comprehensive review presents several limitations. First, a potential publication bias might exist, as unpublished studies or non-English articles were excluded. Second, including various study types and inherent variability in the study quality could introduce heterogeneity and affect the precision of effect estimates. In addition, while comprehensive, the scope of the analysis may not cover every subtopic exhaustively. Quantitative analysis and meta-analysis were not conducted, limiting precise quantitative effect estimates. The complex interplay between AF and DM, potential bias in included studies, and evolving research further contribute to the review's limitations. Nonetheless, these limitations provide a comprehensive understanding of potential challenges and areas for future research in this complex field.

## Conclusions

T2DM and AF represent prevalent, interconnected medical conditions that substantially elevate the risk of severe cardiovascular complications. Diagnosing and managing these diseases pose significant challenges due to their diverse and often subtle clinical presentations, influenced by age, obesity, hypertension, and genetic predisposition. T2DM exerts notable effects on the heart's electrophysiological and structural characteristics, rendering it more susceptible to AF. Nevertheless, the molecular mechanisms underlying this relationship remain incompletely elucidated, necessitating further research to identify potential pharmacological interventions. In addition, T2DM compounds, the clinical course of AF, likely attributable to underlying cardiovascular comorbidities, heightened procoagulant tendencies and altered responses to pharmacotherapies. Optimal care for individuals afflicted with T2DM and AF demands a personalized and comprehensive approach, entailing strategies encompassing anticoagulation, heart rate and rhythm management, lifestyle modifications, and antidiabetic pharmacotherapy. Lifestyle adjustments, such as weight control and regular physical activity, hold promise for enhancing symptom control and glycemic regulation. While specific antidiabetic agents may benefit AF patients, additional empirical evidence is required to validate their effectiveness and safety. The intricate interplay between T2DM and AF necessitates tailored and holistic healthcare. Advancements in unraveling the underlying molecular mechanisms and therapeutic options can potentially ameliorate patient outcomes and overall quality of life. Future research endeavors should prioritize elucidating these intricate molecular processes and conducting rigorous clinical investigations in adherence to established standards and guidelines.
